# Miscellany

**Published:** 1902-02

**Authors:** 


### MISCELLANY.

DEPARTMENT OF HEALTH.—That the work of the physician may be simplified and that school children, who have been quarantined on account of contagious disease, may more readily obtain certificates permitting them to return to school, on and after January 15, 1902, upon receipt of request from the attending physician to remove scarlet fever placards, together with statement that the premises have been disinfected and fumigated; and upon receipt of regulation postal card, in diphtheria cases, that disinfection and fumigation has taken place; and in all other contagious and infectious diseases, we will fill out and deliver to the family, school certificate, in cases where there are school children in the family.

WALTER D. GREENE, M. D., Health Commissioner.

BUFFALO, N. Y., January 10, 1902.

